# CDK9 inhibitor A09-003 overcomes TRAIL resistance via dual Mcl-1 suppression in breast cancer cells

**DOI:** 10.1007/s12672-026-04958-6

**Published:** 2026-05-05

**Authors:** Kyoung Mi Sim, Supyong Hwang, Sojung Park, Eunji Kim, Seak Hee Oh, Inki Kim

**Affiliations:** 1 Convergence Medicine Research Center, ASAN Institute for Life Sciences, ASAN Medical Center, Olympicro 43-gil, Songpagu, Seoul, Republic of Korea; 2 Present Address: Department of Anesthesiology and Pain Medicine, Inha University Hospital, 27 Inhangro, Junggu, Incheon, Republic of Korea; 3 Department of Pediatrics, Asan Medical Center Children’s Hospital, University of Ulsan Medical College, Olympicro 43-gil, Songpagu, Seoul, Republic of Korea; 4 Department of Convergence Medicine, ASAN Medical Center, 88, Olympicro 43-gil, Songpagu, Seoul, Republic of Korea; 5 Department of Convergence Medicine, University of Ulsan Medical College, Olympicro 43-gil, Songpagu, Seoul, Republic of Korea

**Keywords:** Breast cancer, TRAIL, CDK9 inhibitor, Apoptosis, Mcl-1, A09-003

## Abstract

**Background:**

Resistance to tumor necrosis factor–related apoptosis-inducing ligand (TRAIL) is a main limitation in breast cancer therapy. Identifying effective sensitizers is crucial to overcome this hurdle. Cyclin-dependent kinase-9 (CDK-9) inhibition has recently emerged as a potential strategy to enhance apoptotic responses in cancer cells.

**Materials and methods:**

We investigated the effect of A09-003, a novel CDK9 inhibitor, on TRAIL-induced apoptosis and cell signaling in TNBC breast cancer cells. Cell viability was assessed by CellTiter-Glo assays, while apoptotic induction was evaluated using Flow cytometry analysis with Annexin V/PI staining, DNA fragmentation, and caspase activity assay. Protein expression levels of Mcl-1 and death receptors were analyzed via Western blotting and real time PCR. Mechanistic studies explored Mcl-1 protein degradation and transcriptional regulation through CDK-9 inhibition.

**Results:**

A09-003 significantly enhanced TRAIL-induced apoptosis in tested breast cancer cells by increasing death receptor 5 expression, promoting caspase activation, and DNA fragmentation. Mechanistically, A09-003 downregulated Mcl-1 expression through dual pathways: promoting its proteasomal degradation and suppressing transcription via inhibition of CDK9-dependent RNA polymerase II phosphorylation.

**Conclusion:**

A09-003 restores TRAIL sensitivity through Mcl-1 downregulation, identifying CDK-9 as a therapeutic target in resistant breast cancers.

**Supplementary Information:**

The online version contains supplementary material available at 10.1007/s12672-026-04958-6.

## Introduction

The tumor necrosis factor–related apoptosis-inducing ligand (TRAIL) preferentially engages death receptors on cancer cells, specifically DR4 and DR5, while bypassing healthy tissues [1–2]. Activation of these TRAIL receptors initiates a signaling cascade that assembles pro-apoptotic components, including FADD, caspase-8, and FLIP, into a multi-protein complex structure known as the DISC [3–4]. While TRAIL also binds to decoy receptors (DcR1 and DcR2), these lack death domains and thus serve as natural inhibitors by masking TRAIL without apoptosis induction [5–6]. Because of this selectivity, TRAIL has been considered as a tumor-selective apoptosis-inducing agent. However, the therapeutic application of TRAIL has been hampered by the frequent emergence of resistance, even in cancer cells initially responsive to it [7].

This resistance is typically linked to reduced expression of TRAIL-binding death receptors and increased presence of proteins that counteract apoptotic signaling such as Bcl-2, cellular FLIP(c-FLIP), Bcl-xL, and particularly myeloid cell leukemia sequence protein-1 (Mcl-1) [8, 9]. Among these anti-apoptotic proteins, Mcl-1 determines cell fate by blocking mitochondrial-dependent apoptosis signaling [10]. By sequestering pro-apoptotic proteins, Mcl-1 impedes membrane permeabilization in mitochondria and downstream release of apoptosis-inducing factors like cytochrome *c*, thereby suppressing the intrinsic apoptosis signaling [10–11]. The cellular abundance of Mcl-1 is maintained in part through transcriptional control mechanisms by RNA polymerase II which can be activated by cyclin-dependent kinase- 9 (CDK-9) [12–13].

In our previous report, we developed a small molecule CDK-9 inhibitor designated A09-003, which effectively reduces Mcl-1 expression by interfering with CDK9-driven transcription [14]. We also demonstrated that Bay 61-3606, originally described as a spleen tyrosine kinase (Syk) inhibitor, shows inhibitory effect on CDK-9 activity and consequently enhances TRAIL efficacy by suppressing Mcl-1 levels [15].

CDKs, play important roles in regulating cell cycle transitions and cellular survival pathways [16–17]. Inhibitors of these kinases—including flavopiridol, CR8, R-roscovitine, and CDKI-73—have shown pro-apoptotic activity through destabilizing Mcl-1 in various cancer cells [18–21]. However, the detailed mechanism of how Mcl-1 is regulated remains unclear, and highly selective Mcl-1 targeting strategies are needed [22].

In this study, we characterize A09-003 as a CDK9-selective agent capable of reversing TRAIL resistance in breast cancer cells. A09-003 downregulates Mcl-1 at both levels of transcription and post-translation. Our results identify CDK9 as a therapeutic target in Mcl-1-driven cancers.

## Materials and methods

### Cell lines, culture conditions and reagents

The human breast carcinoma cell lines, MDA-MB-231 (cat. no. HTB-26), MDA-MB-436 (cat. no. HTB-130) and MDA-MB-468 (cat. no. HTB-132) were purchased from ATCC (Manassas, VA, USA). Cells were maintained in Dulbecco’s Modified Eagle’s Medium (DMEM; Cytiva, Marlborough, MA, USA, cat. no. SH-30027.01) supplemented with 10% fetal bovine serum (FBS; Gibco, Waltham, MA, USA, cat.no. 16000044), 2 mM L-glutamine (Gibco, cat.no. 25030) and 100 U/mL antibiotics (Gibco, cat.no.15140122). Cultured cells were incubated in 5% CO₂ at 37 °C humidified environment. Recombinant human TRAIL protein (cat.no. PHC 1634) and MG-132 proteasome inhibitor (cat.no. BML-PI102) were purchased from ThermoFisher (Waltham, MA, USA), and Enzo life Sciences (Farmingdale, NY, USA). We used phosphate-buffered saline (PBS, Gibco, cat.no. 10010-023) in each rinsing step.

### Cell viability assesment

A luminescence-based CellTiter Glo assay kit (Promega, Madison, WI, USA, cat.no. G7572) was employed to analyze cell numbers and relative viability. Cells were plated with a density of 9,000 per well in 96-well plate and were pre-exposed to A09-003 for 60 min, followed by TRAIL treatment for additional 24 h. ATP-based luminescence was recorded using a Envision label reader (Perkin Elmer, Shelton, CT, USA) and relative viability was calculated in Prism software. Drug combination synergy calculation was performed by Combination Index and isobologram analysis via Calcusyn software.

### Apoptotic caspase activation assay

For caspase activity assay, cells seeded in 96-well plates received A09-003 (5 µM) with or without TRAIL (1 ng/mL) for 24 h. We used commercial assay kits specific for caspase-3/7 (Promega, cat.no. G8092) and − 8 activity (Promega, cat.no. G8202) per manufacturer’s recommendation, and caspase activity-dependent fluorescent substrate cleavage was measured within ENVISION plate reader. The fluorescence signal intensity and relative activity was calculated using Prism software.

### Apoptosis detection via flow cytometry

To detect apoptosis, Dead Cell Apoptosis Kit (Invitrogen, Carlsbad, CA, USA, cat.no. V13245) was used in flow cytometry analysis. Briefly, cells were seeded and cultured in 6-well dishes at a density of 0.5 × 10^6^ cells per well, followed by compound treatment for 72 h. In next step, cells were detached from plate and washed with PBS with centrifugation at 2,500 x g for 5 min. The collected cells were then suspended again in binding buffer to which propidium iodide (PI; 1 µg/mL) and Annexin V-FITC (0.05 µg/mL) had been added. After incubation in the dark environment at room temperature for 30 min, cell characteristics were quantified by flow cytometry analysis performed in FACS Canto II equipment (BD Biosciences, San Jose, CA).

### Western blotting procedure

Whole-cell extracts were prepared using lysis solution containing protease inhibitor (Roche, Basel, Switzerland, cat. no. 11697498001) and phosphatase inhibitor (Roche, cat. no. 04906837001). Preppared protein extract quantification was performed using a bicinchoninic acid (BCA) analysis method. Equal amount of protein aliquots (20 µg) was resolved by sodium dodecyl sulfate polyacrylamide gel electrophoresis (SDS-PAGE) and resolved proteins were transferred to PVDF membrane. After blocking procedure, the membranes were probed with 1’ antibodies (1:1,000) for 2 h at room temperature or overnight at 4 ◦C, washed, and incubated with secondary antibodies (1:5,000) for 1 h at room temperature. For visualization of protein–antibody complexes, we used ECL-based HRP substrate system (Dynebio, Seongnam, Korea).

Primary antibodies from Cell Signaling Technology (Danvers, MA, USA) were used: anti-DR5(8074), anti-RNA pol II (2629), anti-CDK-9 (2316), anti-phospho-CDK-9 (2549), anti-Mcl-1 (5453), anti-cleaved caspase-3 (9661), anti-PARP(9532), anti- BID (2002), anti- Bcl-xl (2764), anti-c-Myc(18583 S), anti-Myc(2276), anti-phospho ERK (4370), anti- ERK (4695), anti-p-GSK3α/β(9331), anti-GSK3α/β(5676), and anti-phospho Mcl-1 (Thr 163) (14765). The anti-phospho RNA polymerase II (ab70324) antibody was from Abcam (Cambridge, UK). Anti-human ubiquitin antibody was purchased from Santa Cruze Biotechnology (CA, USA, sc-8017). Anti-β actin and anti-DR4 (NB100-56747) antibodies were from Novus (Toronto, Canada).

### Reverse transcription with polymerase chain reaction (RT-PCR) and quantitative real time PCR (qPCR)

Total RNA was purified from cells using an RNA purification Kit (Qiagen, Hilden, Germany, cat. no. 74104), by the manufacturer’s protocol. Reverse transcription reaction was performed with 1 µg total RNA using a cDNA Synthesis Kit (ThermoFisher, cat. no. K1622). After cDNA synthesis, PCR was performed using rTaq Plus 5x PCR premix (Elpis, Seoul, Korea, cat. no. EBT-1321) by the following cycle conditions: Denaturation at 95 ℃ for 30 s, annealing at 60 ℃ for 30 s, and extension at 72℃ for 60 s. A total of 30 cycles were repeated. After reaction, PCR products were separated by gel electrophoresis in a 1.5% agarose gel, and the band images were captured by imaging system.

qPCR was also performed with SYBR Green mix (Applied Biosystems, Waltham, MA cat. no. 4367659). Target gene expression (Mcl-1, c-Myc) was quantified relative to GAPDH, with relative fold-change calculated by the ΔΔCt comparative analysis method. The following primers were used: GAPDH forward 5′ CAGGGCTGCTTTTAACTCTGG-3′, reverse 5′-TGGGTGGAATCATATTGGAACA-3′, Mcl-1 forward 5′-AGGAGGAGGACGAGTTGTAC-3′, reverse 5′-TGATGTCCAGTTTCCGAAGC-3′ and Myc forward 5′-CCCTACCCTCTCAACGACAG-3′, reverse 5′- TTCTTGTTCCTCCTCAGAGTCG-3′.

### Imaging-based viability monitoring

To visualize live versus dead cells, fluorescence-based viability dye kits (Invitrogen, cat. no. L3224) were employed. Cells seeded in 96-well plate (3,000 cells/well) received test agents for 48 h and were stained for viability analysis by manufacturer’s protocol. Image capture and quantification-based analysis were performed using a high-content screening equipment (Operetta, Perkin Elmer, Shelton, CT, USA).

### Gene knockdown by siRNA

Two siRNA duplexes targeting Mcl-1 were synthesized and introduced into MDA-MB-436 cells via lipid-based transfection. Transfections were performed for 72 h in 6-well plates(300,000 cells/well). Efficiency of gene silencing was confirmed by immunoblot analysis. The siRNA sequences targeting Mcl-1 were as follows: siMcl-1-1, sense 5′-AAGUAUCACAGACGUUCUCUU-3′, and anti-sense 5′-GAGAACGUVUGUGAUACUUUU-3′; siMcl-1-2 sense 5′-GAAACGCGGUAAUCGGACUUU-3′, and anti-sense 5′-AGUCCGAUUACCGCGUUUCUU-3′.

### Statistical evaluation

Numerical data from statistical analysis were presented as mean ± SD or SEM. Two-group comparisons were assessed by Student’s *t*-test. Statistical significance were defined as follows: *p < 0.05, **p < 0.01, and ***p < 0.001.

## Results

### A09-003 enhances the reactivity of MDA-MB-436 cells to TRAIL-induced apoptosis

**Fig. 1:**
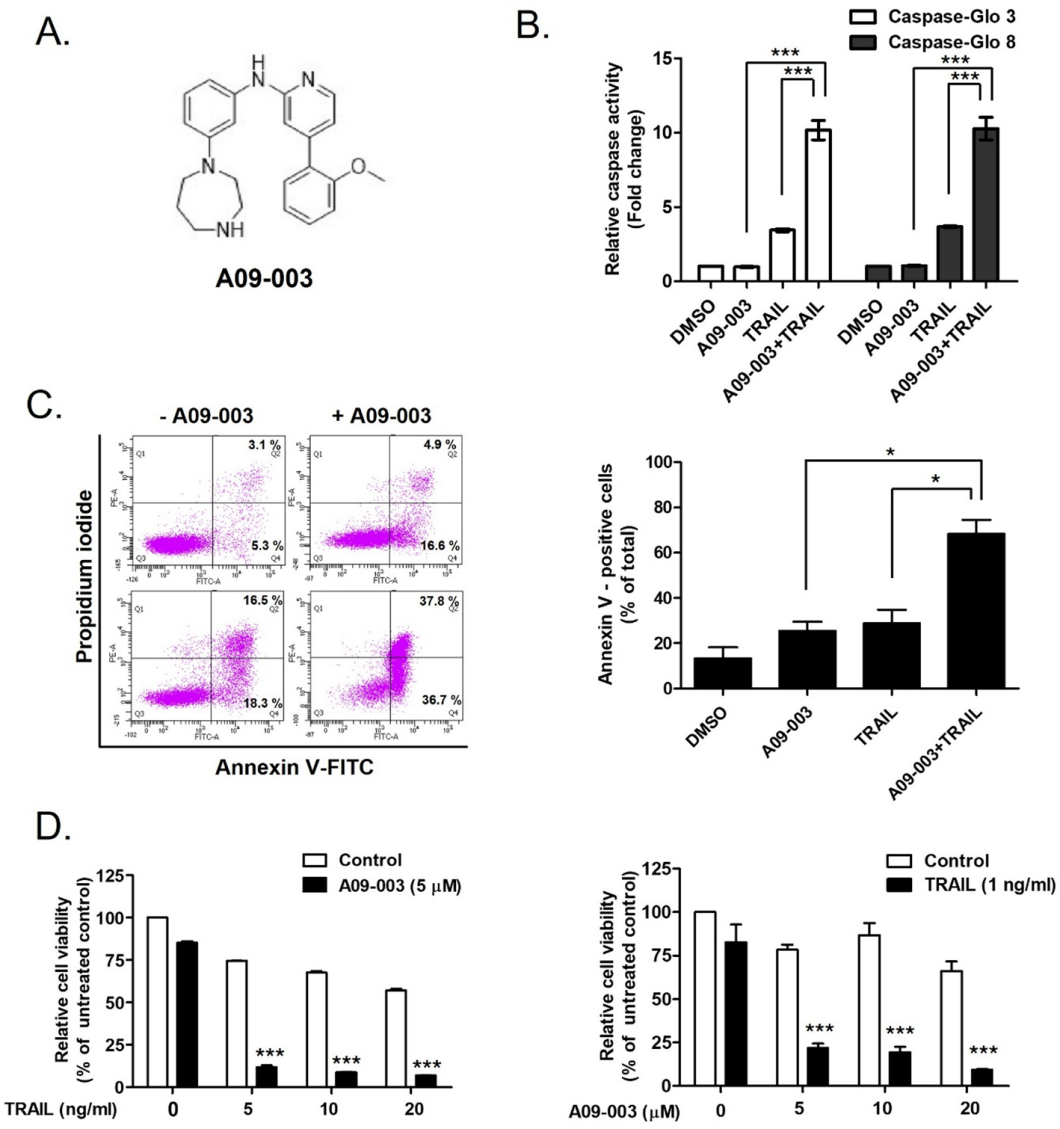
A09-003 augments TRAIL-mediated apoptotic responses in MDA-MB-436 breast cancer cells. **A** Chemical structure of the CDK9 inhibitor A09-003. **B** Cells were treated with A09-003 (5 µM) with or without TRAIL (1 ng/mL), for 24 h, followed by measurement of caspase-3 and caspase-8 activities. Data represent mean ± SD from two independent experiments performed in triplicate. *** P < 0.001 vs. either single agent group. **C** Apoptotic cells were quantified by flow cytometry analysis after 24 h treatment with A09-003 with or without TRAIL. Cells were stained with PI and Annexin V-FITC. Data represent mean ± SD from two independent experiments performed in triplicate. * P < 0.05 vs. either single agent group. **D** Cells were treated with increasing concentrations of TRAIL in the presence or absence of A09-003 (5 µM, left panel) or increasing concentrations of A09-003 with or without TRAIL (1 ng/mL, right panel). Cell viability was measured using the CellTiterGlo assay and the raw luminescent values were normalized to untreated controls. Data represent mean ± SD from two independent experiments performed in triplicate. *** P < 0.001 vs. either single agent

A09-003 is a CDK9 inhibitor previously reported by our group, with the IUPAC name *N-(3-(1*,*4-diazepan-1-yl)phenyl)pyrimidine-2-amine* (Fig. 1A) [14]. A09-003 suppressed cell proliferation and induced apoptosis in a wide range of leukemia cells [14] via Mcl-1 degradation. Since Mcl-1 is a key mediator of TRAIL resistance in a wide range of cancer cells, we investigated whether A09-003 could enhance TRAIL sensitivity in breast cancer cells.

To evaluate this hypothesis, we first examined whether A09-003 enhances TRAIL-induced caspase activation in MDA-MB-436 cells. As shown in Fig. 1B, combined treatment significantly increased caspase-3/7 and caspase-8 activity levels, suggesting enhanced apoptotic signaling. Flow cytometric analysis confirmed this finding, with a marked increase in Annexin-V-positive cells with combined treatment compared to either agent alone (Fig. 1C). Next, we examined the effects of combined treatment of A09-003 with TRAIL on cell proliferation. MDA-MB-436 cells were incubated with the indicated concentrations of both agents for 72 h. As shown in Fig. 1D, combined treatment significantly inhibited cell proliferation, suggesting synergistic effects between A09-003 and TRAIL.

These results show that A09-003 sensitizes MDA-MB-436 cells to TRAIL-induced apoptosis by enhancing caspase activation with cell proliferation reduction, supporting the potential of A09-003 as a TRAIL sensitizer in human breast cancer cells.

**Fig. 2:**
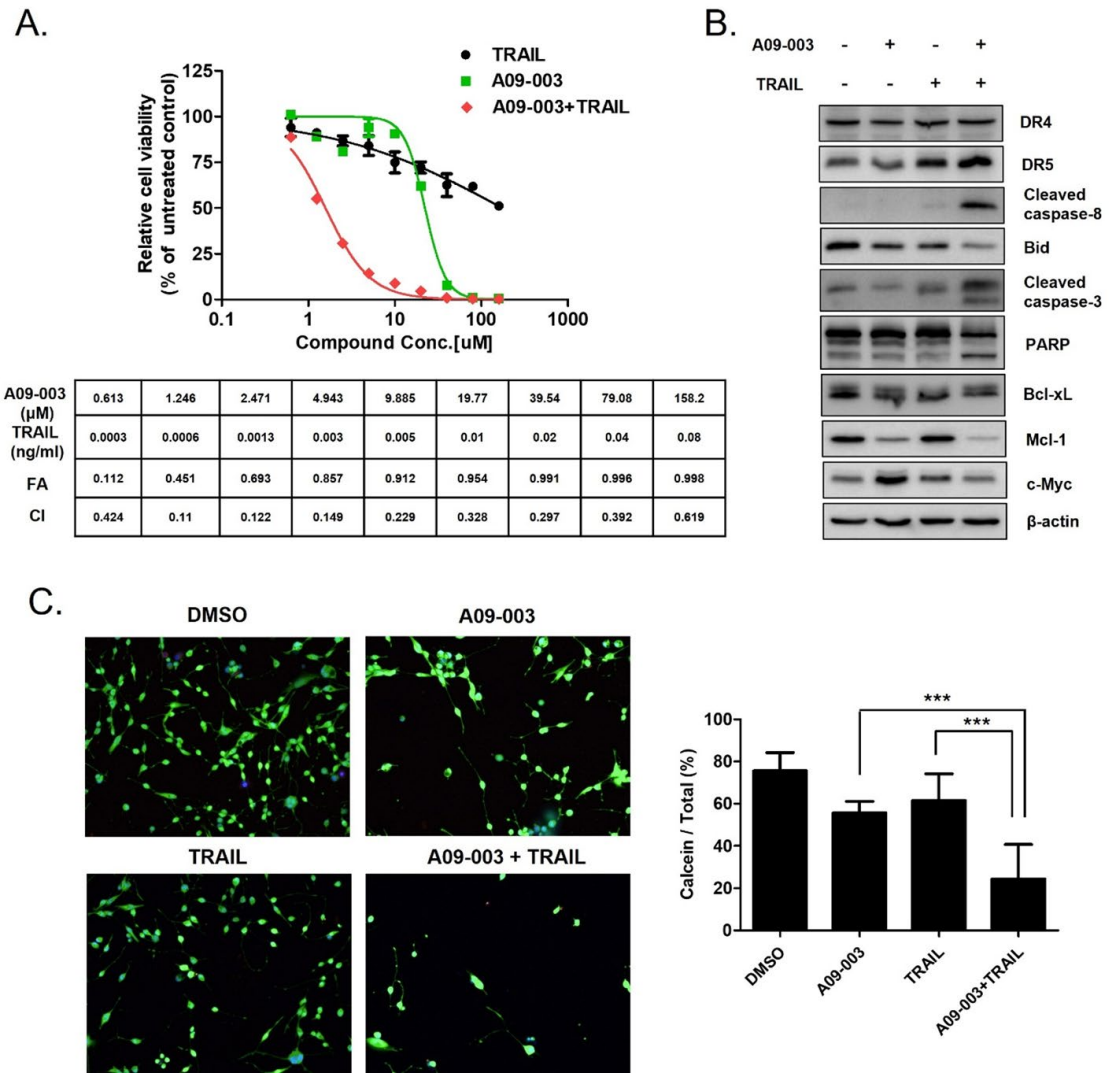
A09-003 and TRAIL synergistically induce apoptosis in breast cancer cells. **A** MDA-MB-436 cells were incubated with the increasing concentrations of A09-003 with or without TRAIL for 72 h. Cell viability was analyzed using the CellTiterGlo assay and normalized to untreated controls. The CI values were calculated across multiple fraction affected (Fa) levels. Each value indicates - CI > 1: antagonistic; CI = 1: additive; CI < 1: synergistic. **B** Western blot analysis of cells which were treated by A09-003 (5 µM) in combination with or without TRAIL (1 ng/mL) for 24 h. **C** Live cell imaging of cells treated for 72 h. The live cells were stained with calcein-AM (green color). Data represent mean ± SD from two independent experiments performed in triplicate. *** P < 0.001 vs. combined treatment

Next, we investigated the synergistic effects of A09-003 and TRAIL combination. Combined treatments were performed using a fixed ratio based on the IC_50_ (half-maximal inhibitory concentration) values of both agents, determined by CellTiterGlo assay. The fraction affected and combination index (CI) were calculated to measure drug interactions (antagonistic, additive, or synergistic). Across most tested concentrations in MDA-MB-436 cells, CI values ranged from 0.11 to 0.62, indicating synergistic interactions (Fig. 2A). Similar results were observed in two other breast cancer cells, MDA-MB-231 and MDA-MB-468 (Supplemental Fig. S1), confirming synergistic interaction between TRAIL and A09-003.

We next examined protein expression by Western blotting following combined A09-003 and TRAIL treatment. As shown in Fig. 2B and the quantitative densitometric analyses provided in the Supplementary Information 1–2 section, treatment of cells with A09-003 and TRAIL single agent did not significantly alter the expression levels of DR4 or Bcl-2 family proteins such as Bcl-xL. In contrast, DR5 expression was modestly increased by either A09-003 or TRAIL alone, whereas combined treatment resulted in a marked upregulation of DR5, with an approximately 3.8-fold increase compared to the control. These findings suggest that A09-003 selectively enhances TRAIL-mediated apoptotic signaling through DR5 rather than DR4. Consistently, activation of the extrinsic apoptotic cascade was markedly enhanced by the combined treatment. While single-agent treatment induced marginal levels of caspase cleavage, co-treatment with A09-003 and TRAIL led to a robust increase in cleaved caspase-8 (approximately 10-fold) and cleaved caspase-3 (approximately 6-fold) relative to control. Combined treatment also strongly induced PARP cleavage, a well-known marker of apoptosis. These data show that A09-003 sensitizes cells to TRAIL through DR5 upregulation and subsequent activation of the caspase. Notably, A09-003 treatment reduced Mcl-1 expression substantially, suggesting that Mcl-1 downregulation is crucial in overcoming TRAIL resistance in MDA-MB-436 cells.

We confirmed these effects using live-cell imaging to assess cell viability 72 h after treatment. As shown in Fig. 2C, combined treatment reduced cell viability to 51% compared to control, while single agents had minimal effect. These results confirm that A09-003 enhances TRAIL-induced cell death in MDA-MB-436 cells.

### A09-003 downregulates Mcl-1 to enhance TRAIL sensitivity

**Fig. 3:**
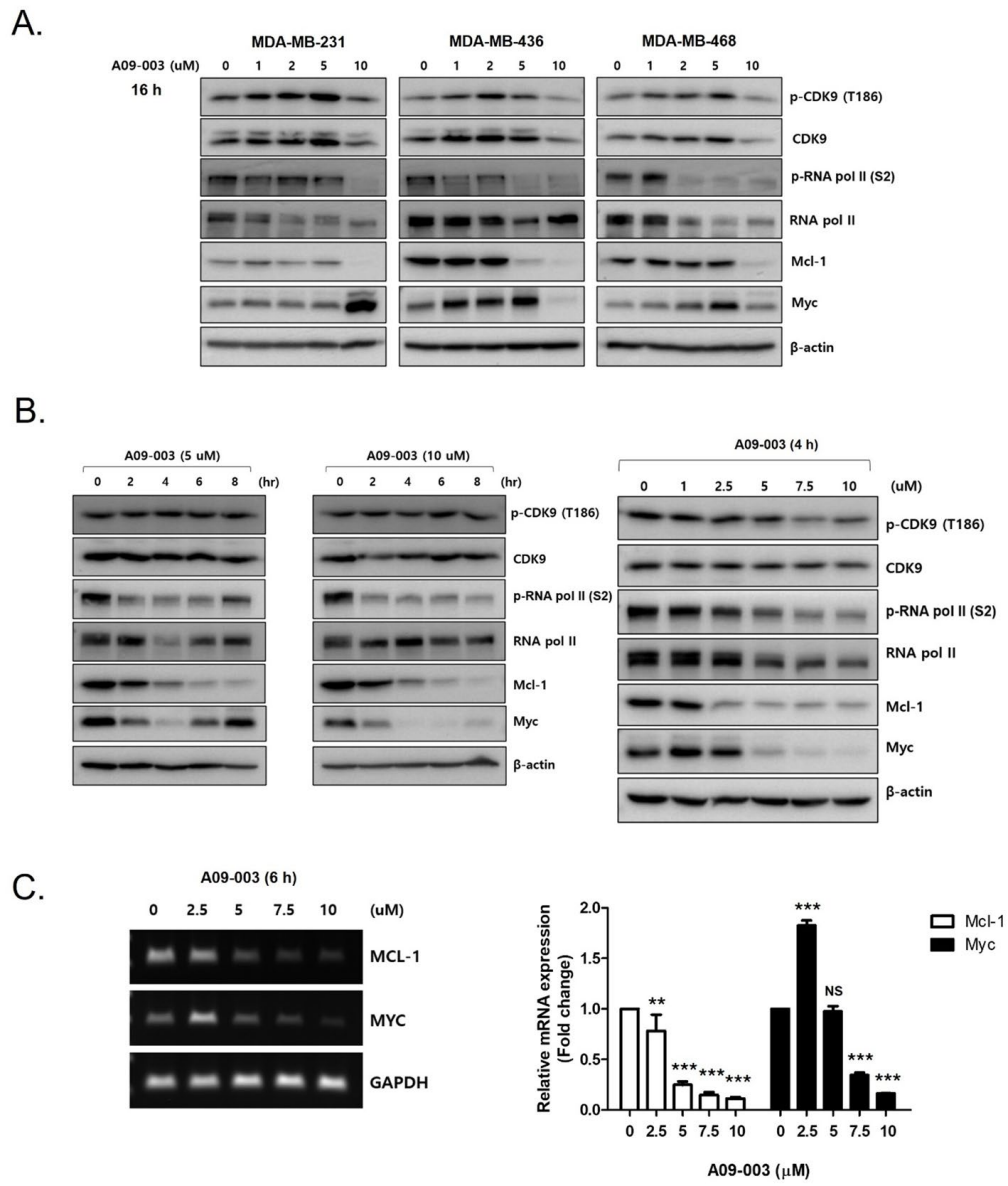
A09-003 inhibits CDK-9 acitivty and reduces Mcl-1 expression. **A** The breast cancer cells were treated with increasing concentrations of A09-003 for 16 h. Western blot analysis shows dose-dependent effects on phospho-CDK9 (Thr186), p-RNA polymerase II (Ser2), Mcl-1, and Myc. β-actin served as a loading control. **B** Time-and, dose-dependent effects in MDA-MB-436 cells. Cells were treated with A09-003 at 5 or 10 µM for the indicated times (left), or for 4 h across a range of concentration (right). **C** A09-003 reduces MCL1 and MYC mRNA levels. Left: RT-PCR showing dose-dependent mRNA reduction after 6 h treatment. GAPDH served as loading control. Right: qPCR quantification normalized to GAPDH. Data represent mean ± SD from two independent experiments. * *P* < 0.05, ** *P* < 0.01, *** *P* < 0.001 vs. untreated control; *NS* not significant

We next examined how A09-003 sensitizes cells to TRAIL. Cells were treated with increasing doses of A09-003 for 16 h and analyzed by Western blotting. As shown in Fig. 3A, A09-003 reduced both phospho-RNA polymerase II levels and Mcl-1 protein expression in breast cancer cells. We confirmed this result in MDA-MB-436 cells using dose-and time-dependent treatment (Fig. 3B) A09-003 progressively reduced phospho-RNA polymerase II, Myc, and Mcl-1 protein levels, indicating effective inhibition of CDK9 (Fig. 3B). Quantitative densitometric analyses corresponding to Fig. 3 are provided in the Supplementary Information.

We also examined the drug effects of A09-003 on Mcl-1 mRNA levels, along with Myc mRNA, another marker of RNA polymerase II activity [23]. MDA-MB-436 cells were exposed to A09-003 for 6 h, and reverse transcription PCR and real-time qPCR were conducted to quantify Mcl-1 and Myc mRNA. As shown in Fig. 3C, A09-003 dose-dependently reduced both Myc and Mcl-1 mRNA levels. These data show that A09-003 reduces Mcl-1 expression at both protein and mRNA levels, demonstrating that CDK9 inhibition effectively targets Mcl-1-driven TRAIL resistance in breast cancer cells.

### A09-003 promotes Mcl-1 degradation through the ubiquitin-proteasome pathway

**Fig. 4:**
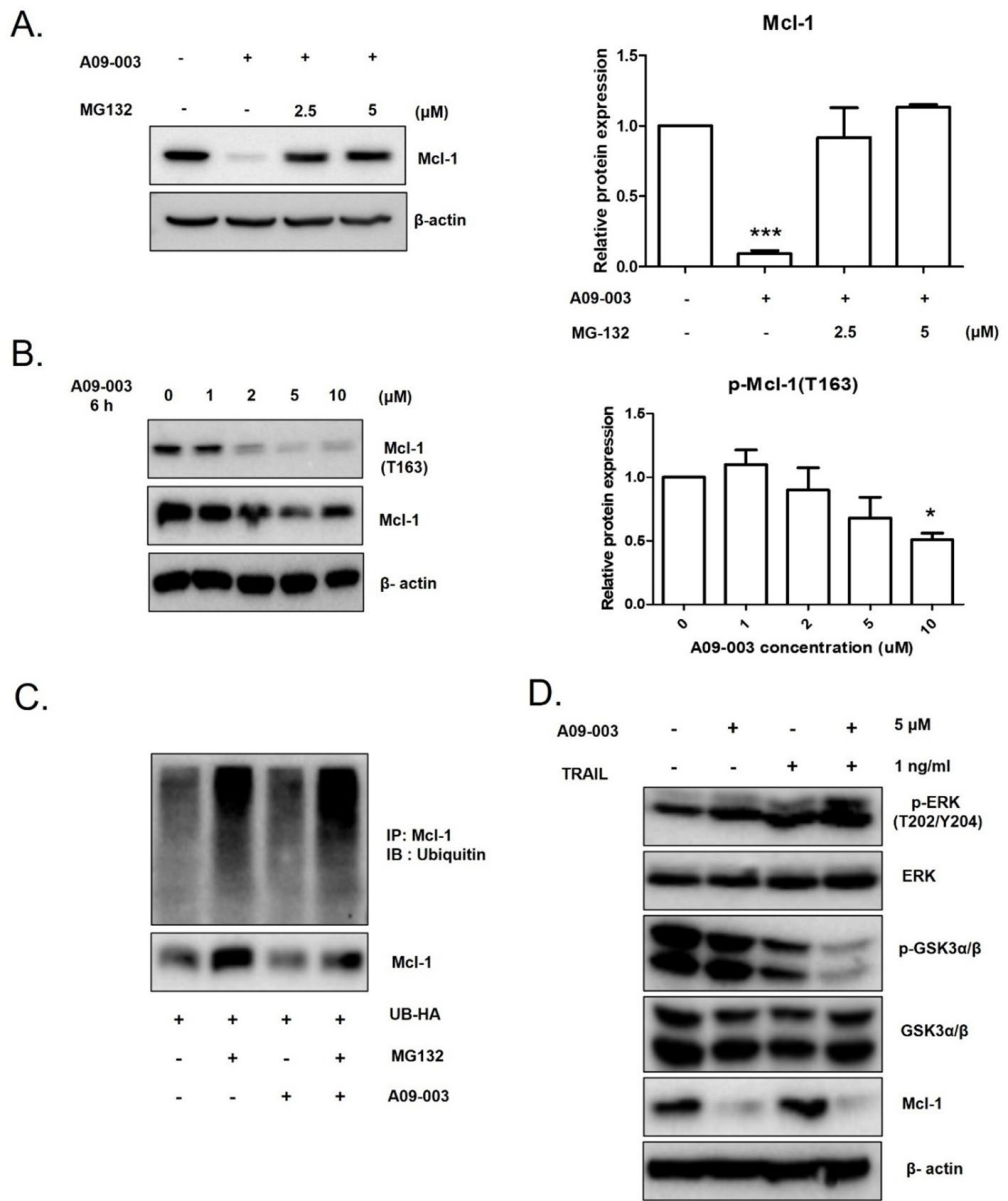
A09-003 promotes Mcl-1 degradation through ubiquitin–proteasome pathway. **A** MDA-MB-436 cells were pretreated with MG-132 (2 µM) for 1 h before A09-003 (2.5 µM, 6 h) treatment. Western blot shows Mcl-1 protein levels. Quantification is shown on the right graph. Data represent mean ± SD from two independent experiments. ***P < 0.001 vs. untreated control. **B** Cells were pretreated with MG-132 (2 µM) for 1 h, then treated with increasing doses of A09-003 for 6 h. Western blot shows dose-dependent effecs on phospho-Mcl-1 (Thr 163) and total Mcl-1. Quantification on the right graph shows phospho-Mcl-1 (Thr163) levels. Data represents mean ± SD from two independent experiments. *P < 0.05 vs. control. **C** Cells were transfected with HA-tagged ubiquitin for 48 h, followed by treatment with A09-003 in the presence of MG-132. Mcl-1 was immunoprecipitated, and ubiquitination was detected by western blot with anti-ubiquitin antibody. **D** Cells were treated with A09-003 (5 µM) and/or TRAIL (1 ng/mL) for 6 h, and cell lysates were subjected to Western blot analysis for the indicated antibodies

We next examined how A09-003 reduces Mcl-1 protein levels. Mcl-1 contains regulatory residues in its N-terminal PEST domain that control phosphorylation, ubiquitination and proteasome-dependent degradation [10]. To test whether A09-003 reduces Mcl-1 through proteasomal degradation, we pretreated cells with the 26 S proteasome inhibitor MG-132. As shown in Fig. 4A, pre-treatment with MG-132 blocked A09-003-induced Mcl-1 reduction, confirming proteasome involvement. We then examined phosphorylation at threonine 163 (Thr163), a conserved MAP (mitogen-activated protein) kinase site within the PEST domain that regulates Mcl-1 stability [24–25]. Phosphorylation at Thr163 is associated with delayed Mcl-1 degradation. Western blot result showed that A09-003 reduced Thr163 phosphorylation, promoting Mcl-1 degradation (Fig. 4B). Next, we assessed Mcl-1 ubiquitination by A09-003 (Fig. 4C). A09-003 alone decreased both ubiquitinated and total Mcl-1, while MG-132 pretreatment caused accumulation of both forms. These results indicate that A09-003 promotes Mcl-1 degradation through the ubiquitin-proteasome-dependent pathway. Quantitative densitometric analysis of ubiquitinated Mcl-1 is provided in the Supplementary Information.

GSK3 also regulates this process by phosphorylating Mcl-1 at serine 159 (Ser159), a site closely associated with Thr163 [26]. Phosphorylation at Ser21 (by GSK-3α) or Ser9 (by GSK-3β) inhibits GSK3 activity [26]. As shown in Fig. 4D and the densitometric analysis provided in the Supplementary Information, p-GSK-3α/β levels were reduced to approximately 0.2-fold relative to the control following combined treatment with A09-003 and TRAIL. suggesting increased activation of GSK3. This enhanced GSK3 activity likely promoted Mcl-1 phosphorylation at Ser159 and subsequent degradation. Though previous studies have linked MEK (mitogen-activated protein kinase kinase)/ERK signaling to GSK3 phosphorylation, our data showed no significant changes in ERK phosphorylation following A09-003 treatment, indicating an ERK-independent pathway. Quantitative densitometric analyses corresponding to Fig. 4D are provided in the Supplementary Information. Our results demonstrate that A09-003 promotes Mcl-1 degradation by reducing Thr163 phosphorylation, enhancing ubiquitination, and activating GSK3 independently of the ERK.

### Knockdown of Mcl-1 gene mimics A09-003-induced sensitization to TRAIL

**Fig. 5:**
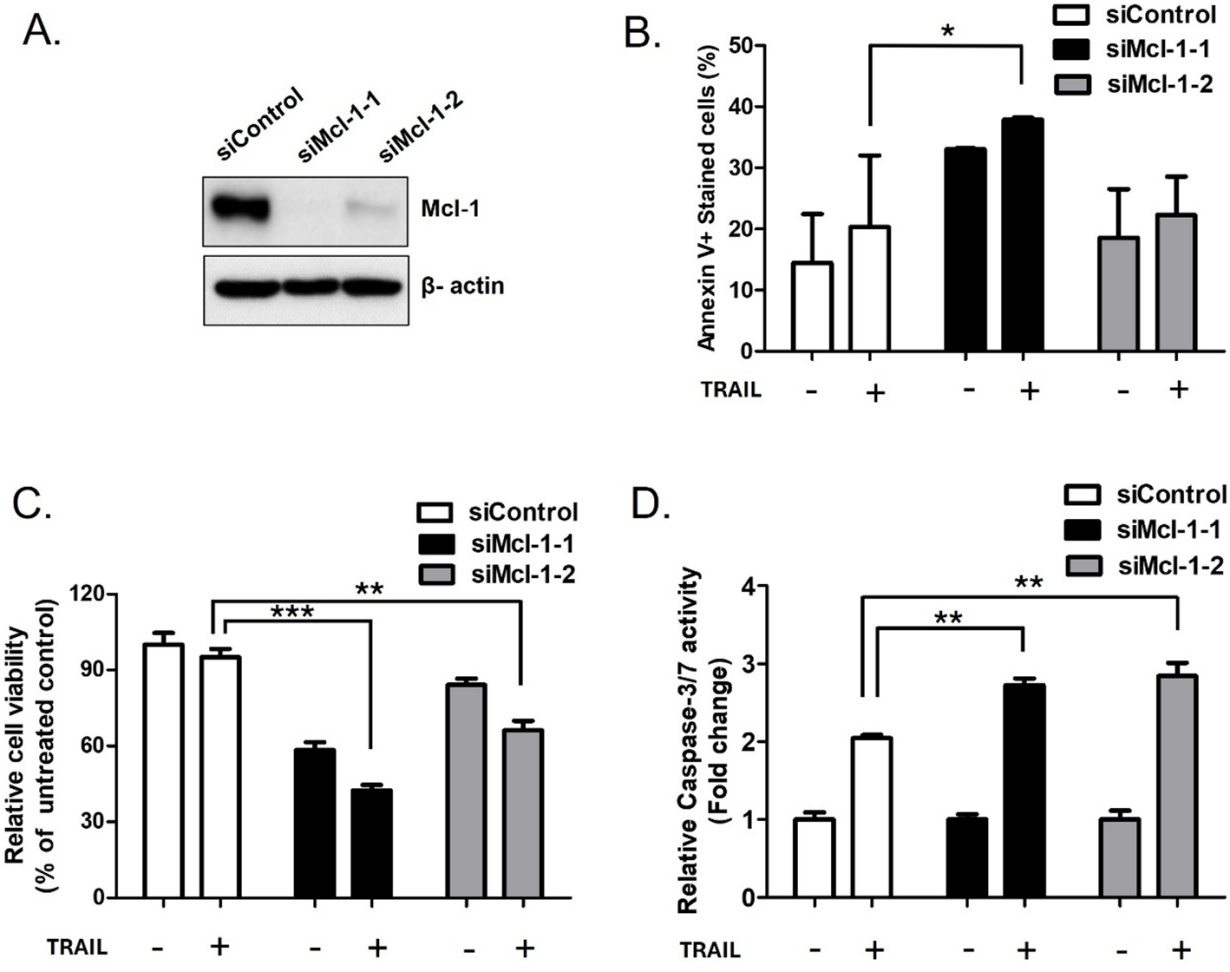
Knockdown of Mcl-1 gene sensitizes MDA-MB-436 cells to TRAIL. **A** MDA-MB-436 cells were transfected with two independent siRNAs targeting MCL1, or non-targeting control. After 48 h, Mcl-1 knockdown was confirmed by Western blotting. β-actin served as a loading control. **B** Transfected cells were treated with TRAIL (1 ng/mL) for 24 h and stained with Annexin V-FITC and PI. Graph shows percentage of Annexin V-positive cells. **C** Transfected cells were treated with TRAIL (1 ng/mL) for 72 h. Cell viability was measured by CellTiterGlo. **D** Caspase-3/7 activity was measured after 24-hour TRAIL treatment (1 ng/mL). Data in panels (**B**–**D**) represent mean ± SD from two independent experiments, each conducted in duplicate. *P < 0.05, ** *P* < 0.01, *** *P* < 0.001 relative to control

Since A09-003 reduced Mcl-1 expression, we tested whether Mcl-1 knockdown alone could enhance TRAIL sensitivity. We transfected MDA-MB-436 cells with siRNA targeting Mcl-1. Western blotting confirmed efficient Mcl-1 knockdown (Fig. 5A). Silencing of Mcl-1 gene alone sensitized cells to TRAIL-induced apoptosis, as shown by reduced cell viability, increased Annexin V staining, and enhanced caspase-3/7 activity (Fig. 5, B-D). These results show that Mcl-1 downregulation is critical for TRAIL sensitivity. Thus, A09-003 sensitizes breast cancer cells to TRAIL through Mcl-1 degradation.

## Discussion

We showed that A09-003, a CDK9 inhibitor, sensitizes breast cancer cells to TRAIL-induced apoptosis through Mcl-1 downregulation and degradation. Combining A09-003 with TRAIL enhanced caspase-3/7 and caspase-8 activity with reduction of cell viability in MDA-MB-436 cells (Fig. 2). These results identify A09-003 as a potent TRAIL sensitizer in breast cancer cells with high Mcl-1 levels.

Mcl-1 is an established target in cancer [27]. Consistent with our finding using A09-003, previous studies have linked CDK9 inhibition to Mcl-1 degradation and the subsequent initiation of apoptosis in cancer cells. For example, wogonin inhibits CDK9 activity and suppresses Mcl-1 transcription level in cancer cells [28]. CDK9 inhibitors are also in clinical development for hematologic malignancies such as acute myeloid leukemia [29].

A09-003 reduces Mcl-1 through two mechanisms : proteasome-dependent degradation of Mcl-1 via ubiquitination (Fig. 4) and transcriptional suppression by CDK9 activity and resulting RNA polymerase II inhibition (Fig. 3). A09-003 reduces Thr163 phosphorylation in Mcl-1, promoting its degradation. GSK3 activation also contributes to Mcl-1 degradation independently of the ERK pathway. This contrasts with the previous studies showing ERK in Mcl-1 regulation [30], suggesting alternative upstream kinase regulation in Mcl-1 stability control.

Our study also demonstrated that knockdown of Mcl-1 gene sensitized cells to TRAIL (Fig. 5), confirming that Mcl-1 downregulation is sufficient to sensitize cells to TRAIL (Fig. 5). Similar observation in other cancer studies support Mcl-1 as a critical regulator of TRAIL sensitivity [28, 31].

While our study used TNBC cells, the mechanisms identified are not limited to TNBC. Mcl-1 overexpression is found across breast cancer subtypes and related with poor prognosis and resistance in various spectrum of breast cancers. Recent studies showed that CDK9 inhibitors could effectively reduce Mcl-1 levels in a wide range of breast cancer subtypes with similar mechanisms.

As Mcl-1 regulates both intrinsic and extrinsic apoptotic signaling, A09-003 may enhance sensitivity to various chemotherapeutic agents as well as TRAIL. Previous reports demonstrated that CDK9 inhibitors could induce synergistic cell death with doxorubicin and taxanes through Mcl-1 suppression with overcoming resistance against chemotherapeutic agents. Future study will evaluate the efficacy of A09-003 in combination with general cancer therapeutic regimens in Mcl-1-driven resistance.

In summary, A09-003 sensitizes breast cancer cells to TRAIL through dual Mcl-1 suppression mechanism. This dual mechanism supports combining CDK9 inhibition with TRAIL in Mcl-1-driven cancers. Further studies using in vivo models of breast cancers such as xenograft should evaluate this strategy in TRAIL-resistant tumors.

## Supplementary Information

Below is the link to the electronic supplementary material.

Supplementary Material 1.

## Data Availability

Data available on request from the corresponding author.
