# Supplementary material for: CDK9 inhibitor A09-003 overcomes TRAIL resistance via dual Mcl-1 suppression in breast cancer cells
Source: Discov Oncol. 2026 May 5;17:932. doi: 10.1007/s12672-026-04958-6 (PMC13287533; doi:10.1007/s12672-026-04958-6)

Supplementary Information file for manuscript

1. **Synergistic interaction between A09-003 and TRAIL in breast cancer cells.**
2. **Densitometric analysis- quantitative bar graph for Western blot data**
3. **Uncropped blot images for each western blotting data & RT-PCR agarose gel original image**

- **In all preparation procedures of Western blotting, the blots were cut before antibody hybridization.**
- **Quantitative western blot analyses were performed by normalizing the signal intensity of each target protein to β-actin.**
- **All western blot densitometric analyses were carried out using ImageJ software (NIH, USA).**

**1. Synergistic interaction between A09-003 and TRAIL in breast cancer cells.**


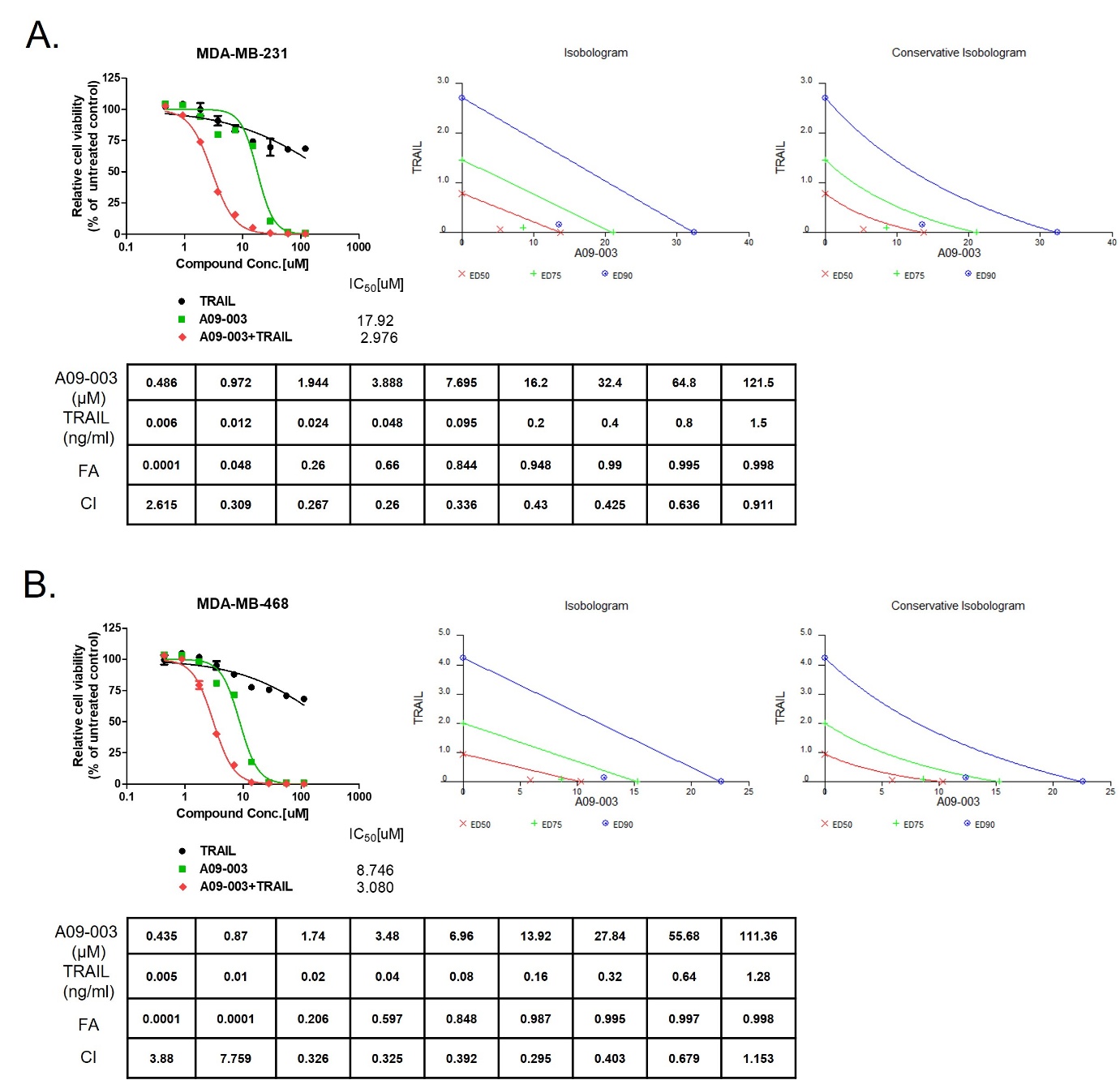


Supplementary Fig. 1 Synergistic interaction between A09-003 and TRAIL in breast cancer cells.
(A, B) MDA-MB-231(A) and MDA-MB-468(B) cells were treated with the indicated concentrations by combination of A09-003 with or without TRAIL for 72 h. Cell viability was measured using CellTiter-Glo assay. Left panel shows dose-response curves with calculated IC_50_ values. Middle and right panels show isobologram and conservative isobologram analysis by Chou-Talalay method.Tables below show fraction affected (FA) and combination index (CI) values at different concentration combination. CI < 1 denotes synergistic interaction. Data represent mean ± SD.

**2. Densitometric analysis- quantitative bar graph for Western blot data**

- 1. **Quantitative bar graph for Western blot in Figure 2B**


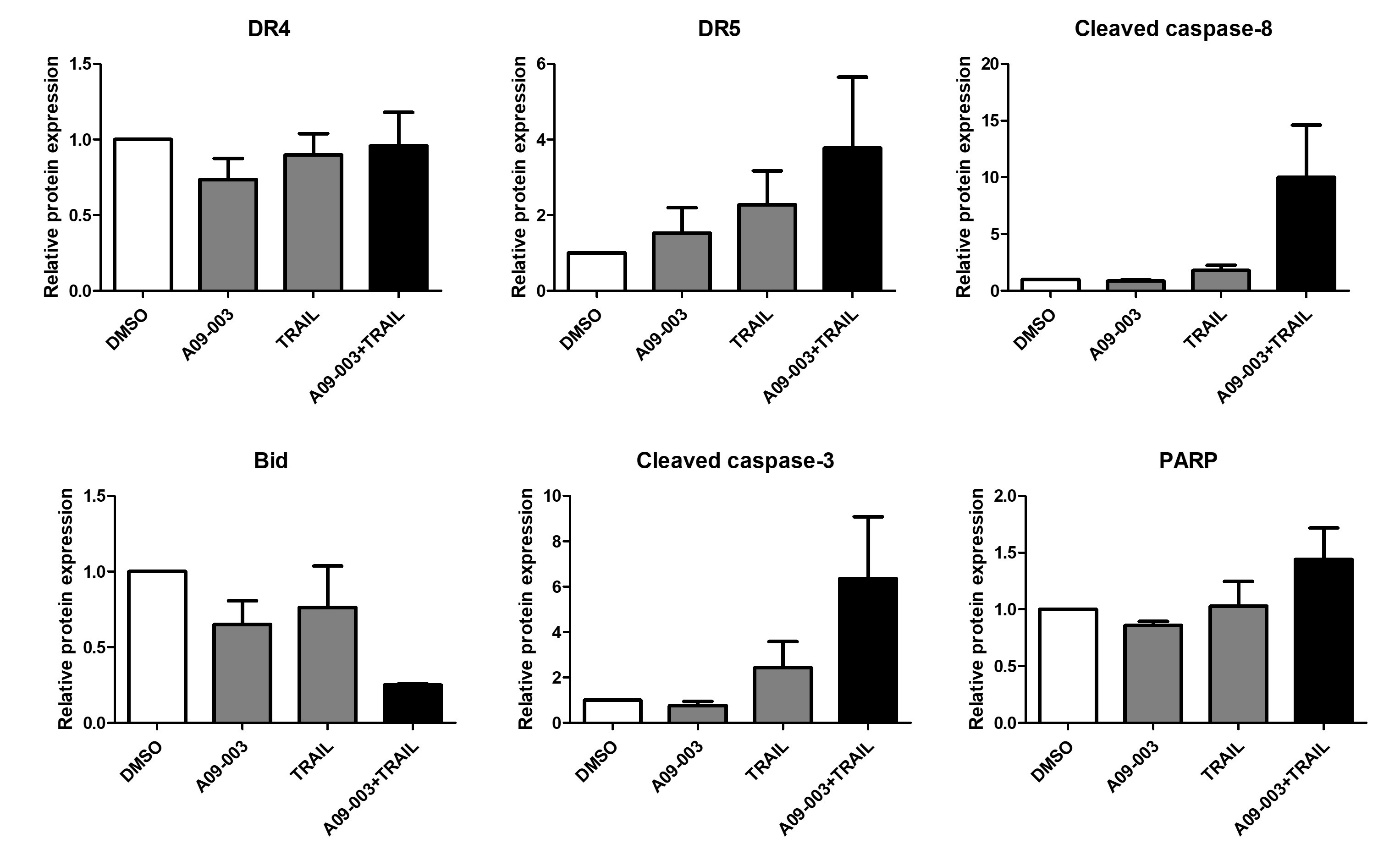

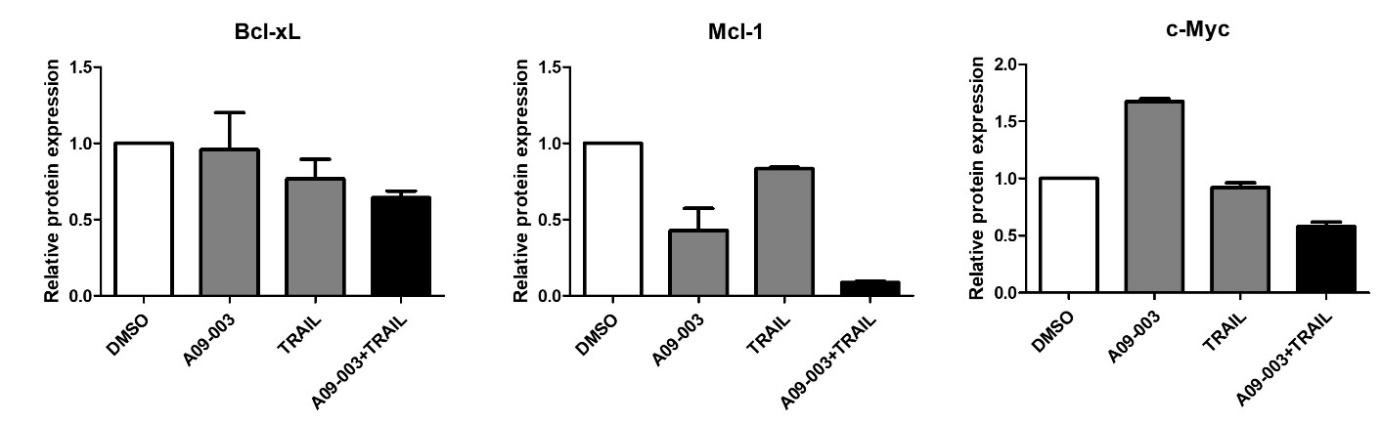


All data represent mean ± SEM from two independent biological experiments.

- 1. **Quantitative bar graph for Western blot in Figure 3A**

MDA-MB-231


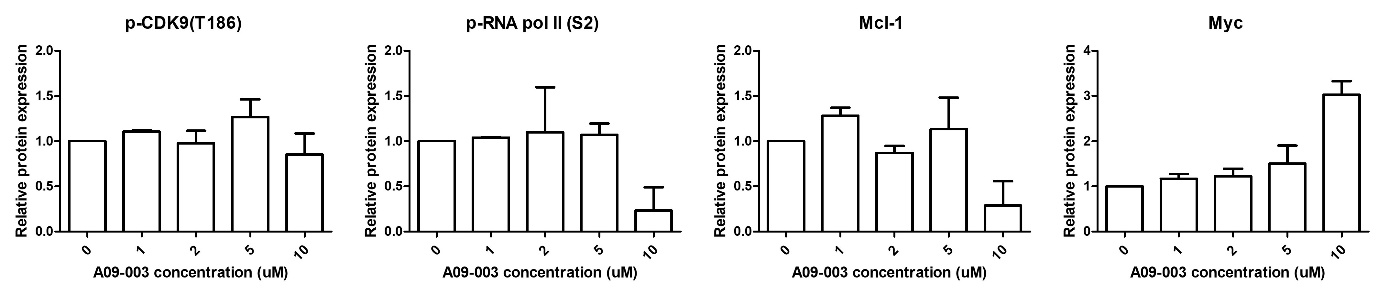


MDA-MB-436


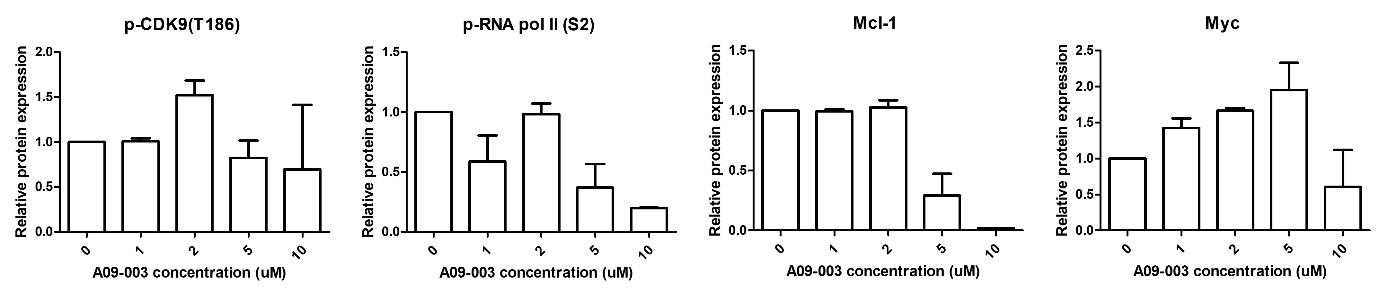


MDA-MB-468


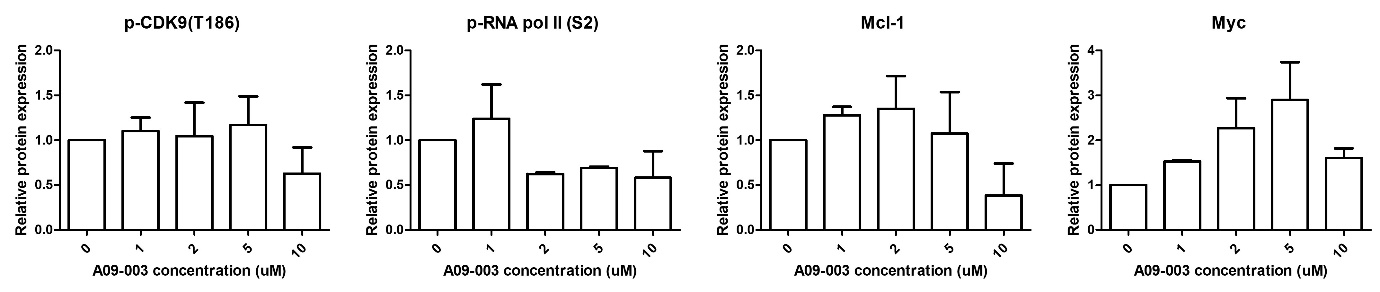


All data represent mean ± SEM from two independent biological experiments.

- 1. **Quantitative bar graph for Western blot in Figure 3B**

A09-003 (5 μM)


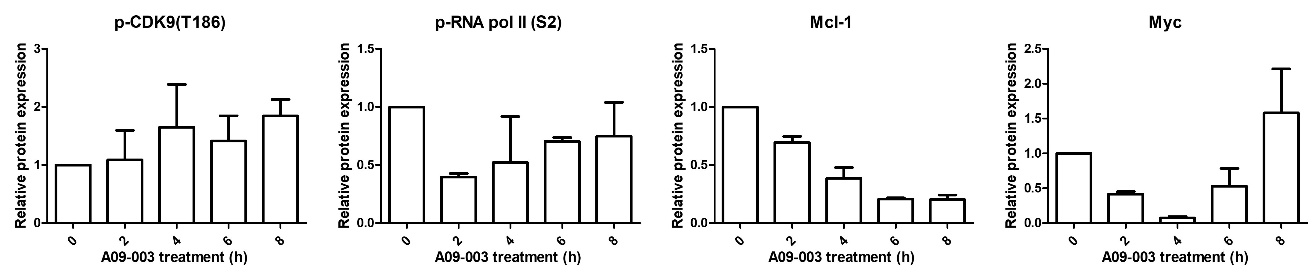


A09-003 (10 μM)


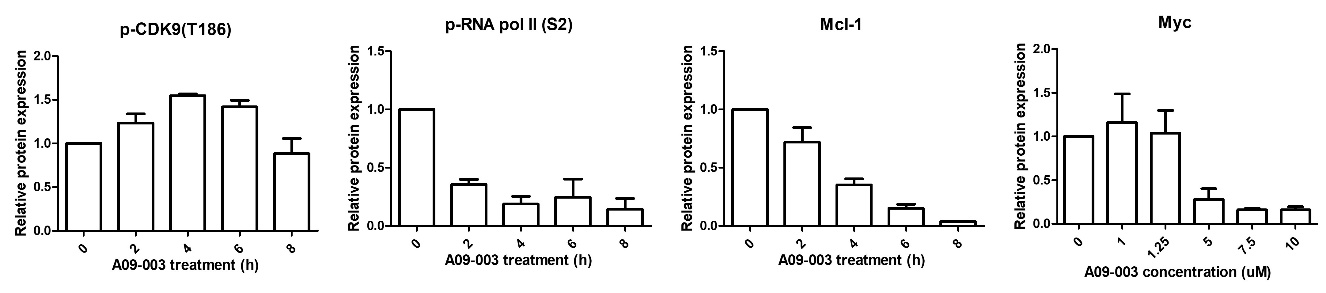


A09-003 (4 h)


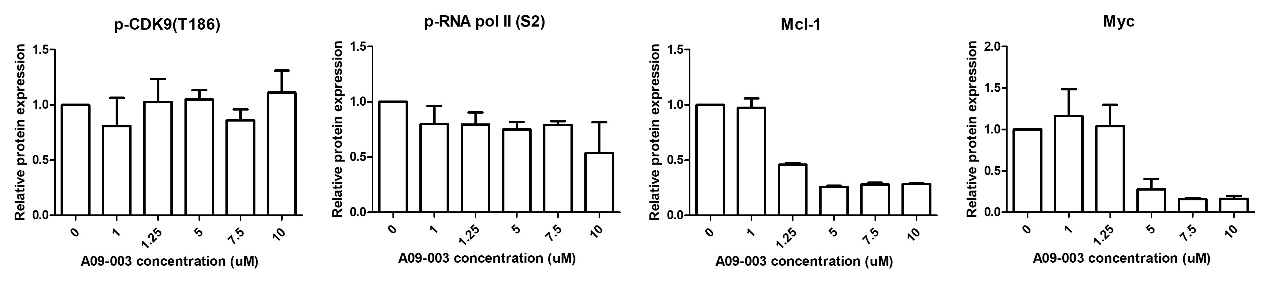


All data represent mean ± SEM from two independent biological experiments.

- 1. **Quantitative bar graph for Western blot in Figure 4**

Figure 4 C


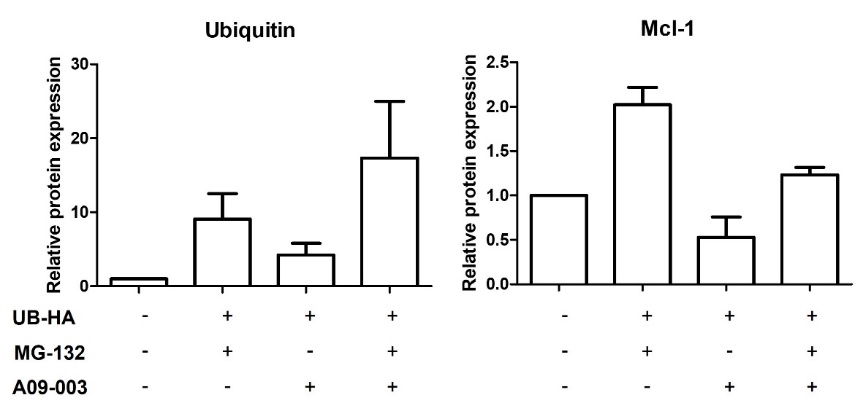


Figure 4 D

**
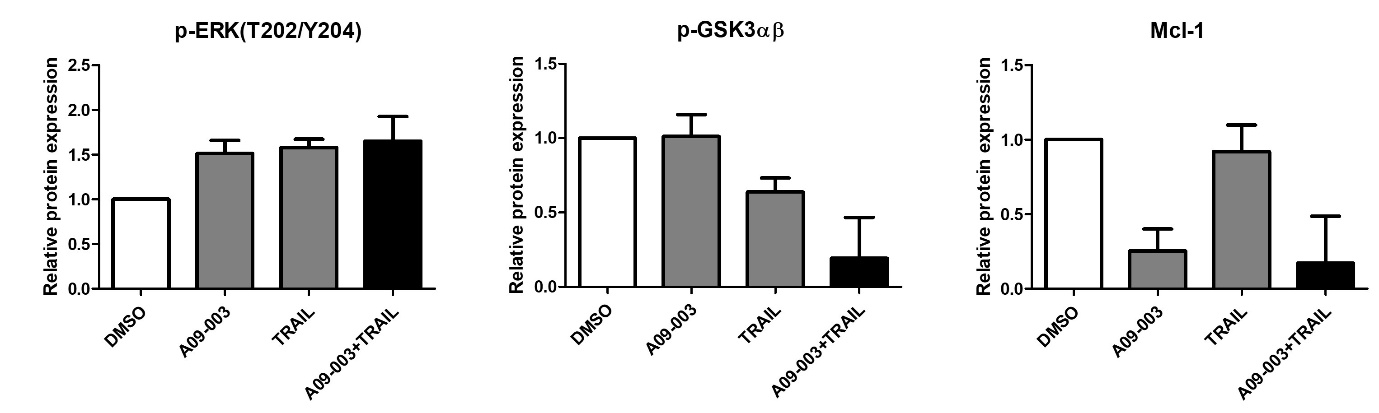
**

All data represent mean ± SEM from two independent biological experiments.

- 1. **Quantitative bar graph for Western blot in Figure 5**


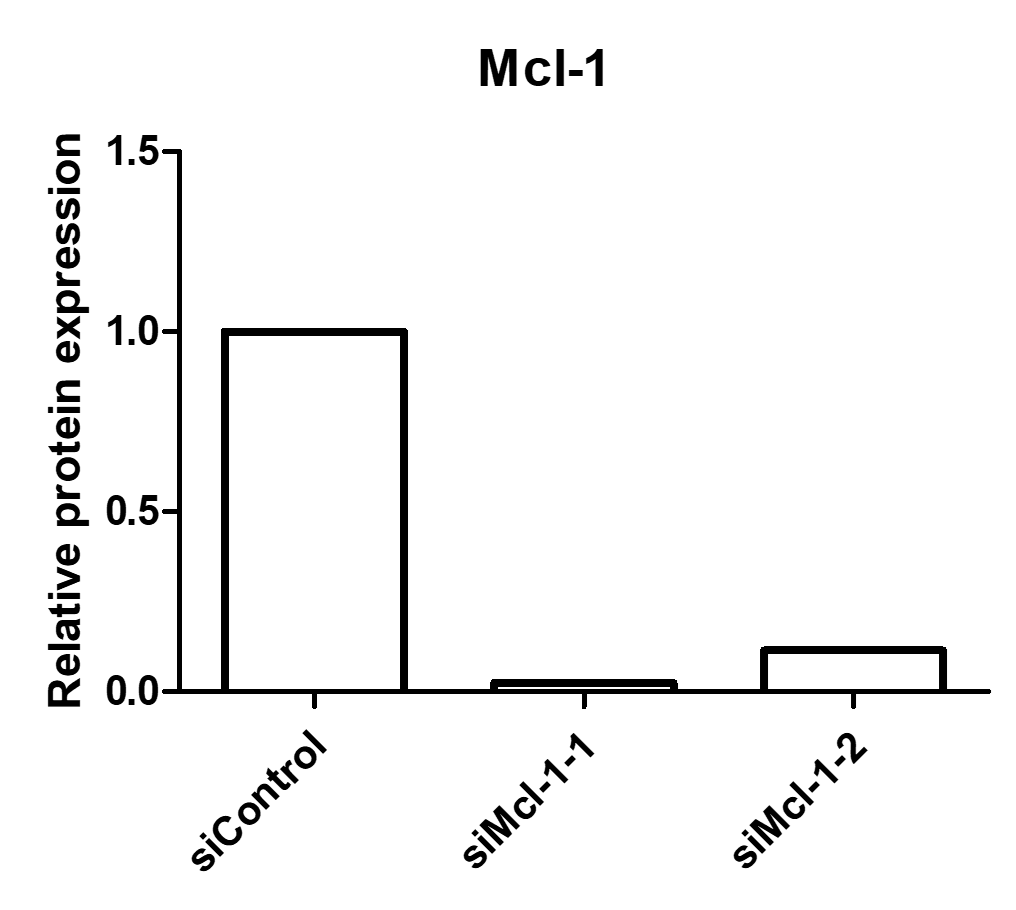


**3. Uncropped blot images for each western blotting data**

**3-1. Original images of western blots for Figure 2B**


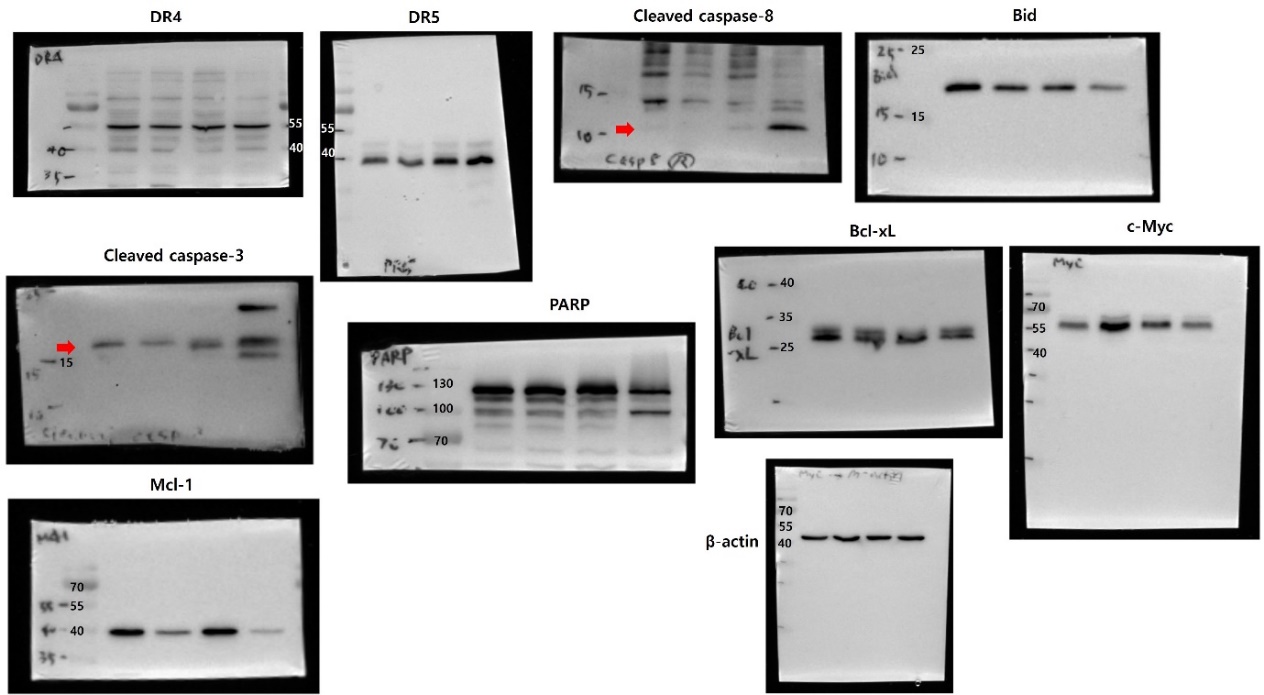


- 1. **Original images of western blots for Figure 3A.**


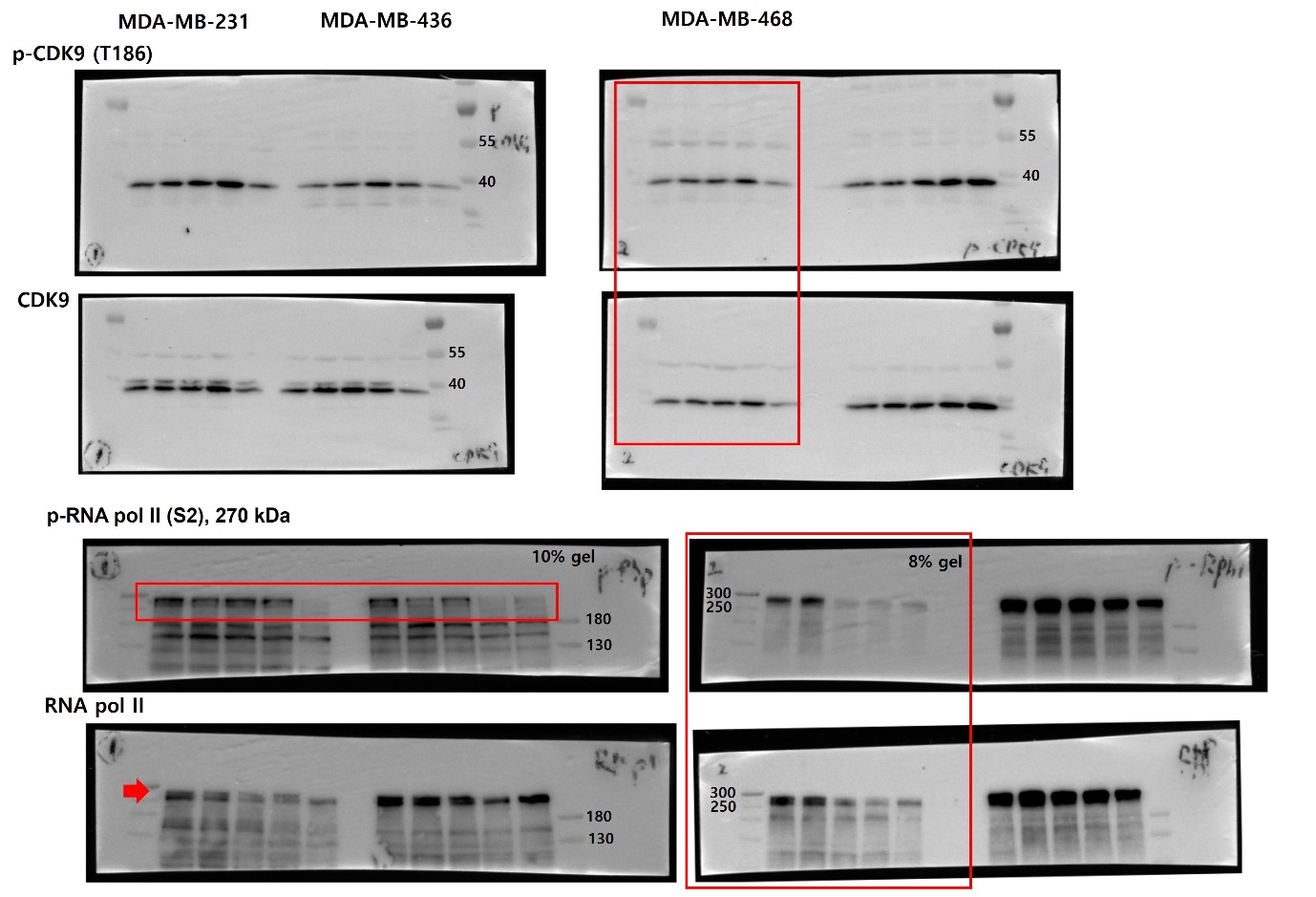


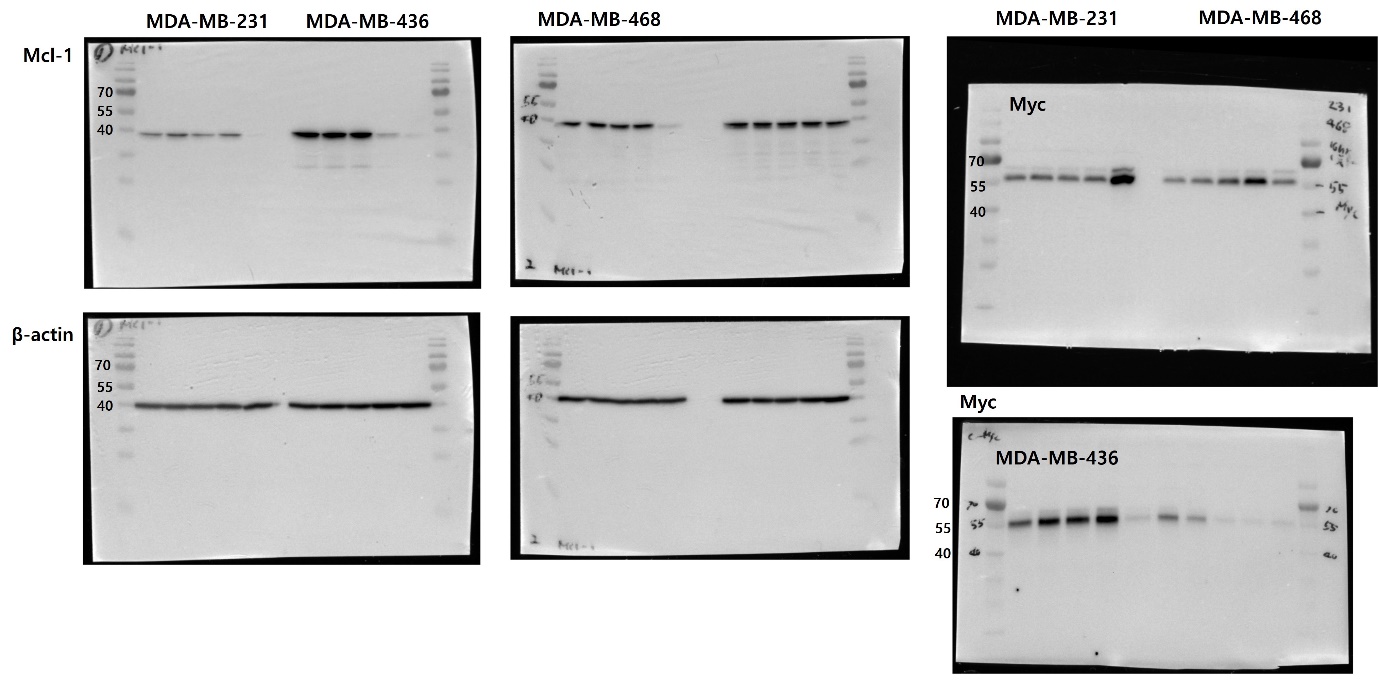
 The right part band image is utilized in other experiments, serving no purpose as a reference in this context.

- 1. **Original images of western blots for Figure 3B.**
- **Left panel**


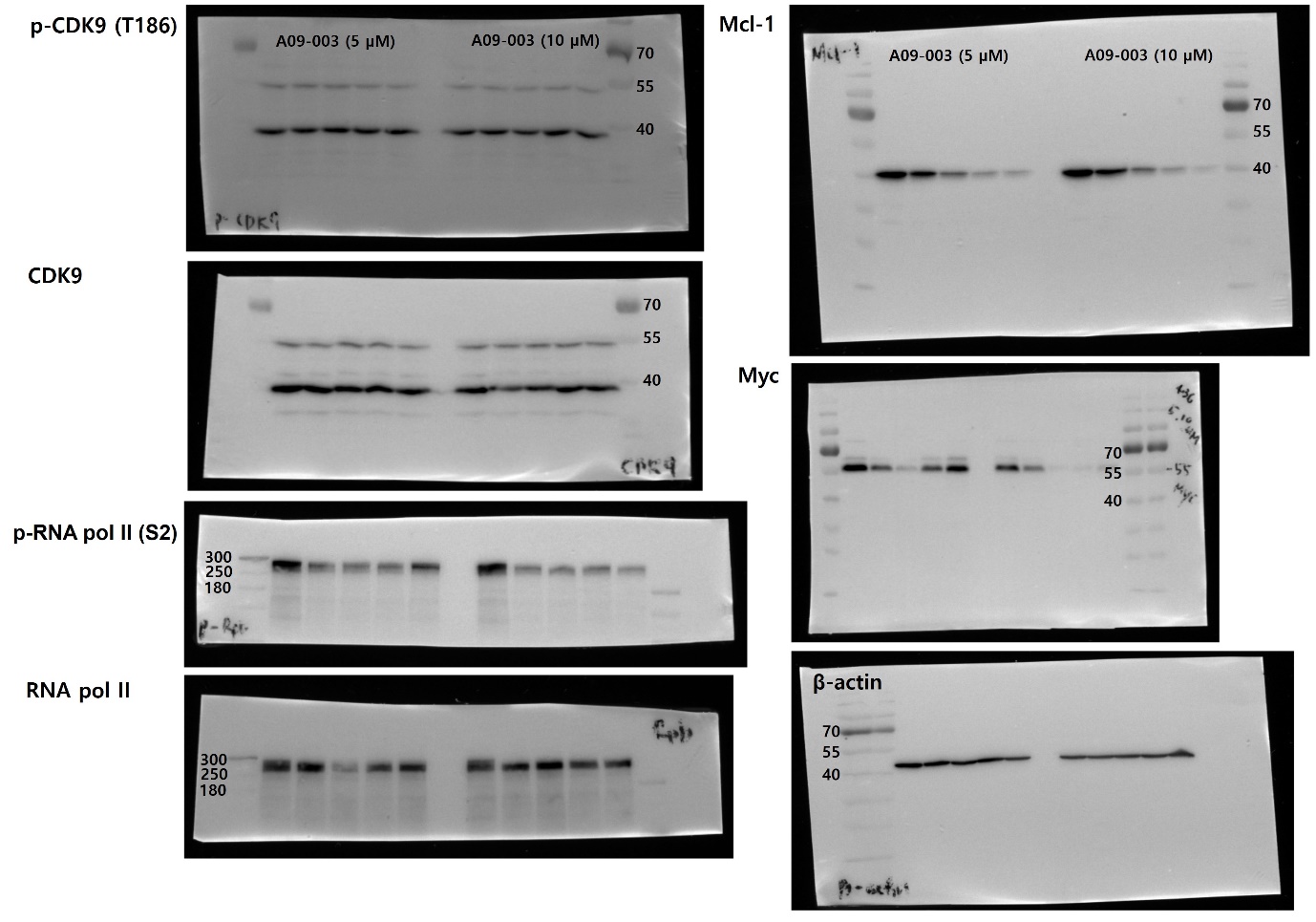


- **Right panel**


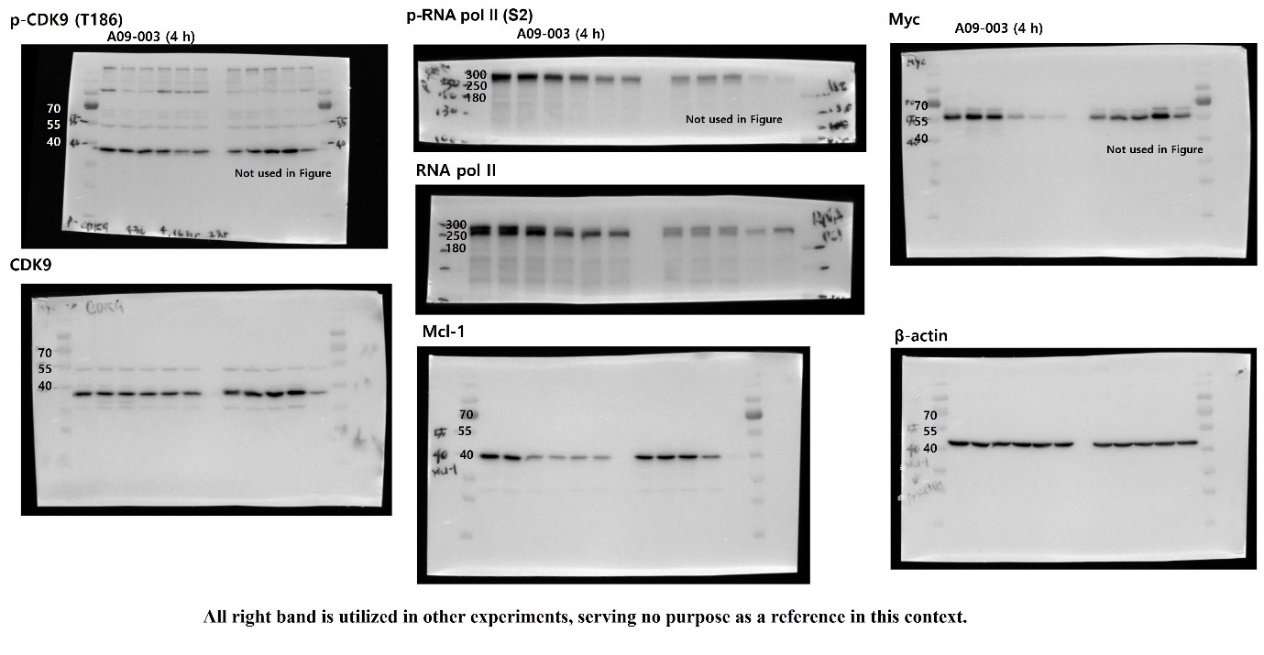


- 1. **Original images of conventional PCR for Figure 3C.**


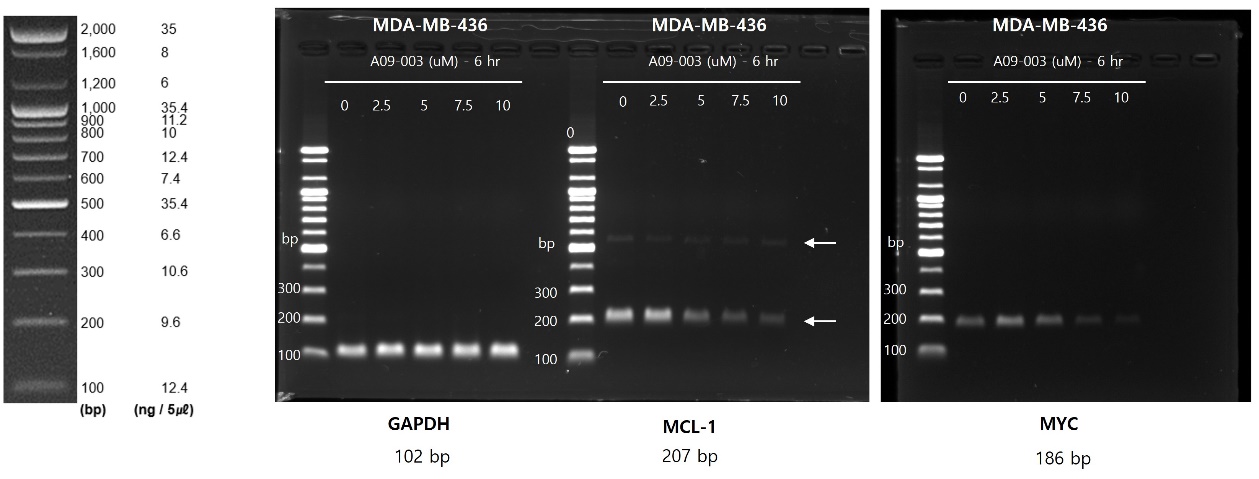


- 1. **Original images of western blots for Figure 4.**


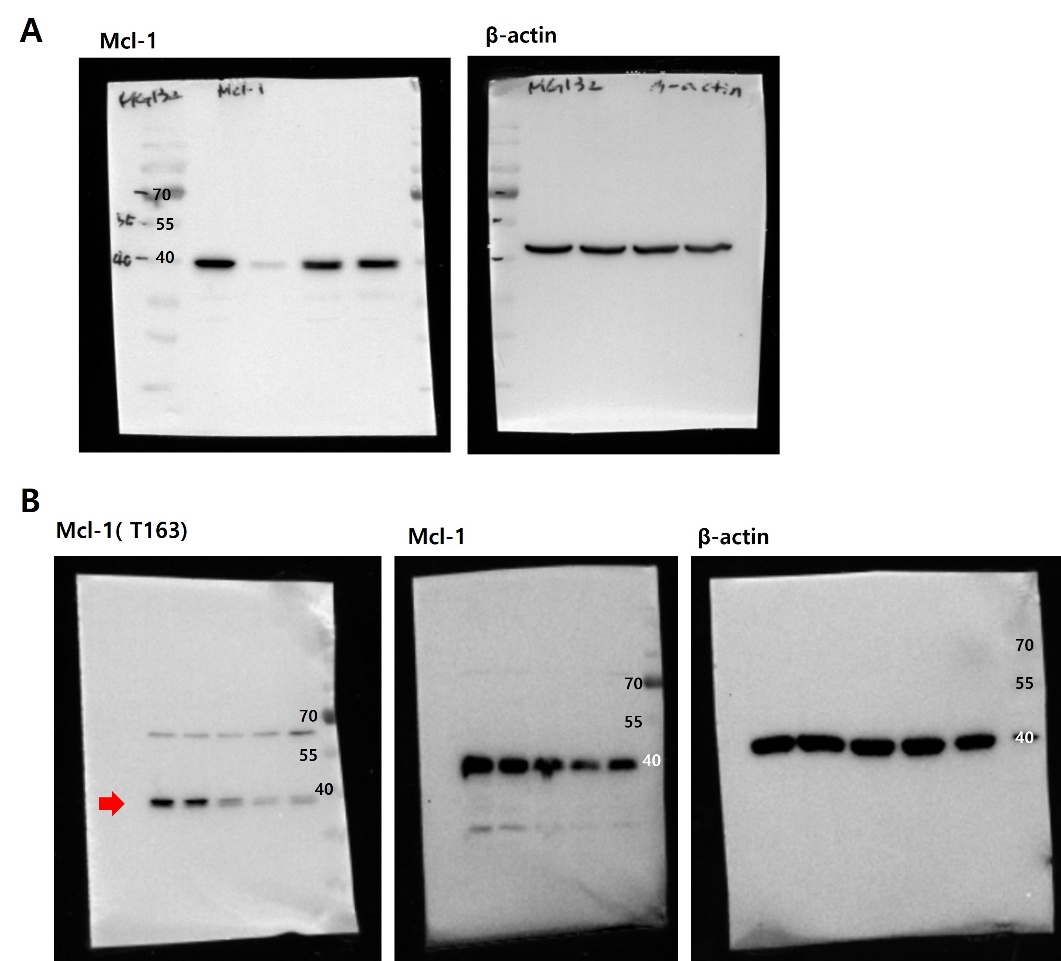


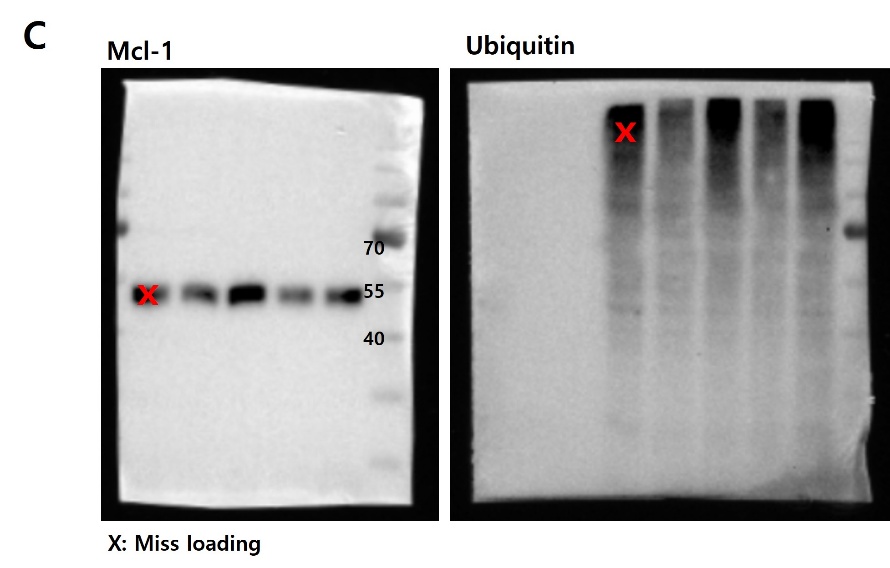


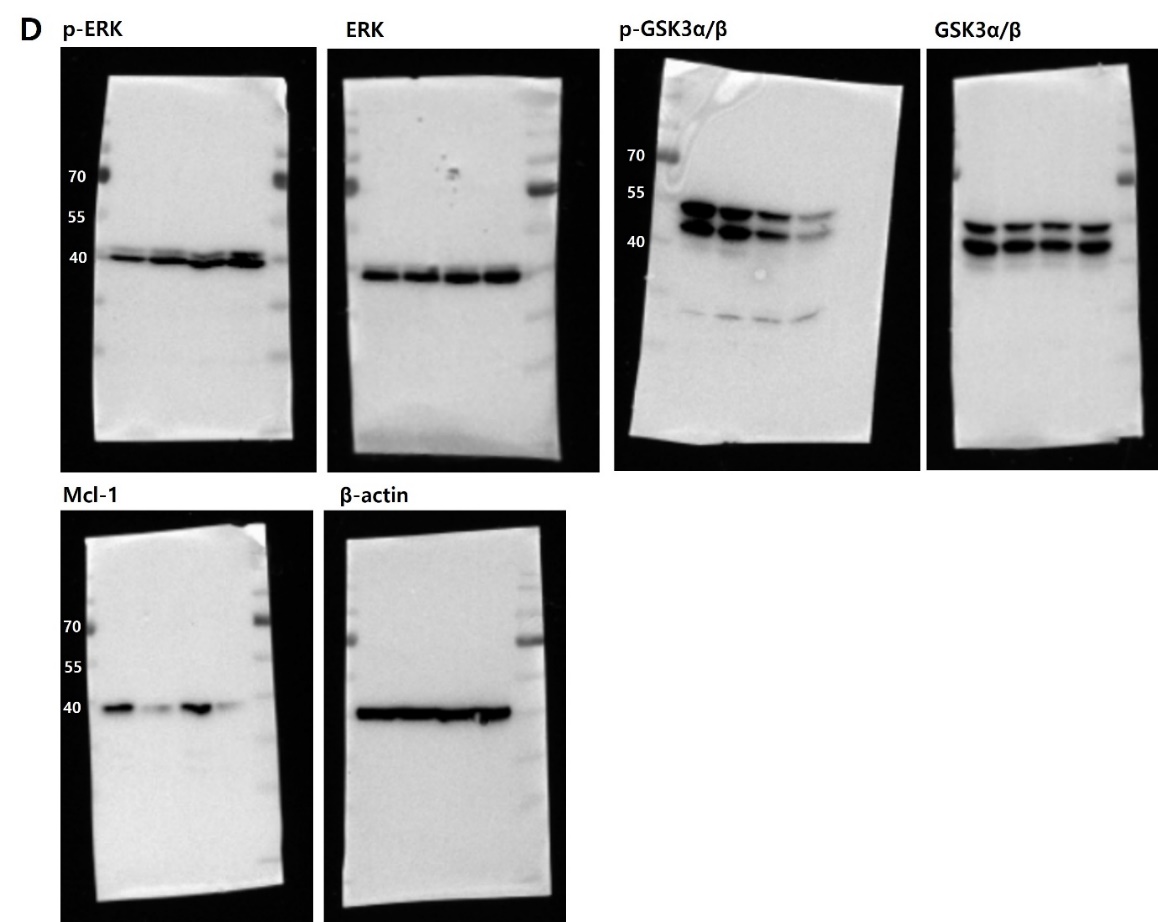


- 1. **Original images of western blots for Figure 5.**


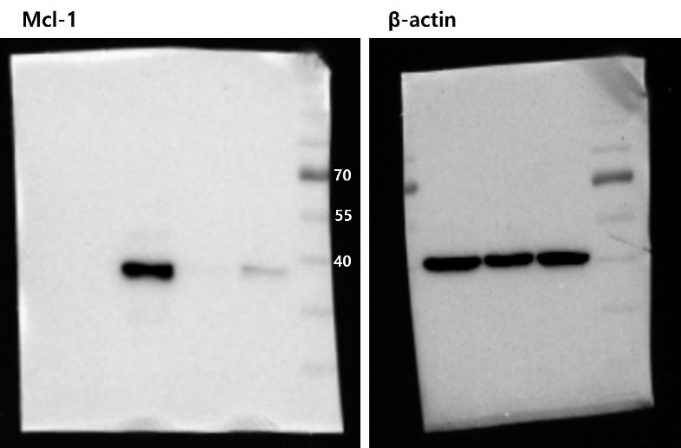

Supplement: Supplementary file 1 — Supplementary Material 1. [file 12672_2026_4958_MOESM1_ESM.docx]
